# Health & Demographic Surveillance System Profile: The Magu Health and Demographic Surveillance System (Magu HDSS)

**DOI:** 10.1093/ije/dyv188

**Published:** 2015-09-24

**Authors:** Coleman Kishamawe, Raphael Isingo, Baltazar Mtenga, Basia Zaba, Jim Todd, Benjamin Clark, John Changalucha, Mark Urassa

**Affiliations:** ^1^National Institute for Medical Research, Mwanza Centre, Mwanza, Tanzania and; ^2^Department of Population Health, London School of Hygiene and Tropical Medicine, London, UK

**Keywords:** Demography, fertility, mortality, migration, verbal autopsy, sero survey, INDEPTH network, Magu, Tanzania

## Abstract

The Magu Health and Demographic Surveillance System (Magu HDSS) is part of Kisesa OpenCohort HIV Study located in a rural area of North-Western Tanzania. Since its establishment in 1994, information on pregnancies, births, marriages, migrations and deaths have been monitored and updated between one and three times a year by trained fieldworkers. Other research activities implemented in the cohort include: sero surveys which have been conducted every 2–3 years to collect socioeconomic data, HIV sero status and health knowledge attitude and behaviour in adults aged 15 years or more living in the area; verbal autopsy (VA) interviews conducted to establish cause of death in all deaths encountered in the area; Llnking data collected at health facilities to community-based data; monitoring voluntary counselling and testing (VCT); and assessing uptake of antiretroviral treatment (ART). In addition, within the community, qualitative studies have been conducted to address issues linked to HIV stigma, the perception of ART access and adherence.

In 2014, the population was over 35 000 individuals. Magu HDSS has contributed to Tanzanian estimates of fertility and mortality, and is a member of the INDEPTH network. Demographic data for Magu HDSS are available via the INDEPTH Network’s Sharing and Accessing Repository (iSHARE) and applications to access HDSS data for collaborative analysis are encouraged.

Key Messages

- Magu HDSS is a platform for HIV epidemiological studies, using a well-established identification procedure for linking individuals’ health status and family data with social, economic and health facilities data.
- Observed deaths rates among HIV-positive adults were 15 times higher than those among HIV-negative adults, and nearly half of deaths were associated with HIV/AIDS.
- Mortality among under-fives has declined significantly during the period 1994–2011.
- Due to impact of ARV, a substantial mortality decline has been observed among HIV-positive individuals, at a time when there is little or no mortality decline in HIV-negative individuals.

## Why was the HDSS established?

Magu HDSS was established in 1994 by the Tanzania-Netherlands Support Program on AIDS (TANESA) as one the vital components of the Kisesa Open Cohort HIV Study. The Kisesa Open Cohort was set up to monitor the causes, spread and impact of the HIV/AIDS epidemic in the general population. The Kisesa Open Cohort is the second oldest HIV community cohort study in Africa and is responsible for monitoring population dynamics, HIV, access to HIV treatment and the impact of the treatment.

The underlying rationale for the establishment of Magu HDSS was to create a community-based surveillance system to measure child and adult mortality, fertility and mobility in the general population. Magu HDSS data have been used to measure individual and household impacts of HIV and antiretroviral therapy (ART). They have also been used to assess the leading causes of death and changes in family structure due to the adult mortality. Furthermore the HDSS has been an important source of data for district planning at ward and district levels. Since late 1995 several community interventions have been introduced to promote safer sexual behaviour. The Magu HDSS is a member of the INDEPTH network [http://www.indepthnetwork.org] and the ALPHA network [http://www.lshtm.ac.uk/eph/psd/alpha].

## Where is the HDSS area?

**Figure 1.**
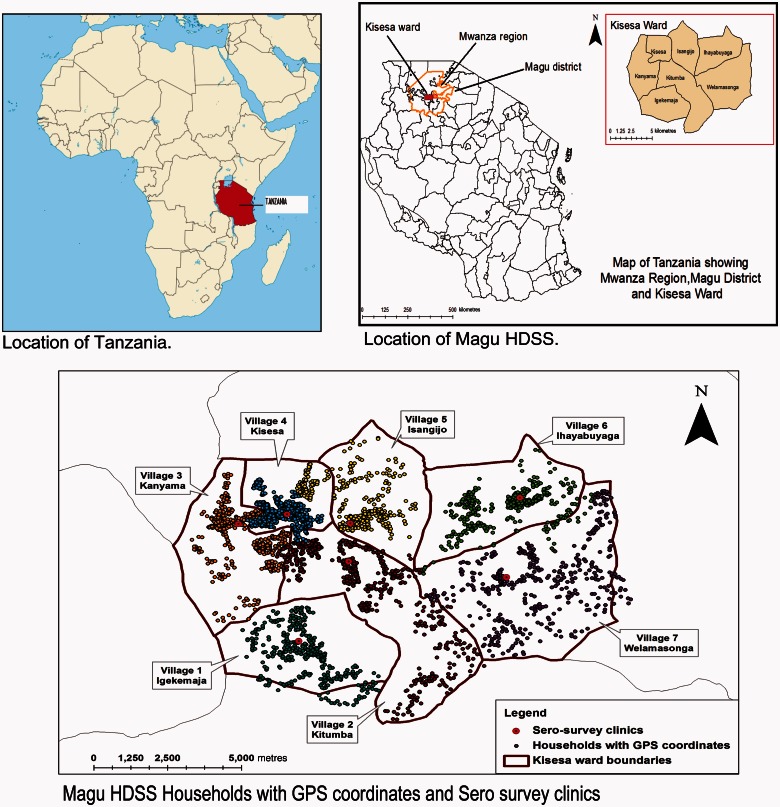
Map showing the Magu Health and Demographic Surveillance area.

In 1994, Magu HDSS consisted of seven villages wholly located in Kisesa ward. Kisesa ward is one of the 31 wards of Magu district (Figure 1). Magu is one of seven districts of Mwanza Region in North-Western Tanzania. The Magu HDSS study area lies 20 km east of Mwanza, the region’s capital city, and is located along the main road to neighbouring Kenya.

Farming is the main source of income (97%) and commonly grown crops are cotton, cassava, paddy, maize and sweet potatoes, and petty trade of products such as milk, tomatoes, maize and rice is common. Within the Magu HDSS area there are four Government-owned health facilities (all providing maternal and child health services) and three private dispensaries. There are also 15 primary and three secondary schools in the area.

## Who is covered by the HDSS and how often have they been followed up?

Magu HDSS covers all residents of the Kisesa ward. Data collection began in 1994 with the baseline census, which involved listing all individuals living in the HDSS’s geographical area. The baseline survey led to the creation of demographic household cards. Households are self-defined as ‘a group of people living together in the same compound and who regularly eat together from the same pot’. The cards contain a list of all individuals living in a particular household, with each household member having their own unique HDSS number. HDSS numbers are never re-assigned to another household member following a member’s exit and, if a member leaves and returns to the same household, they get their old HDSS number back.

**Figure 2.**
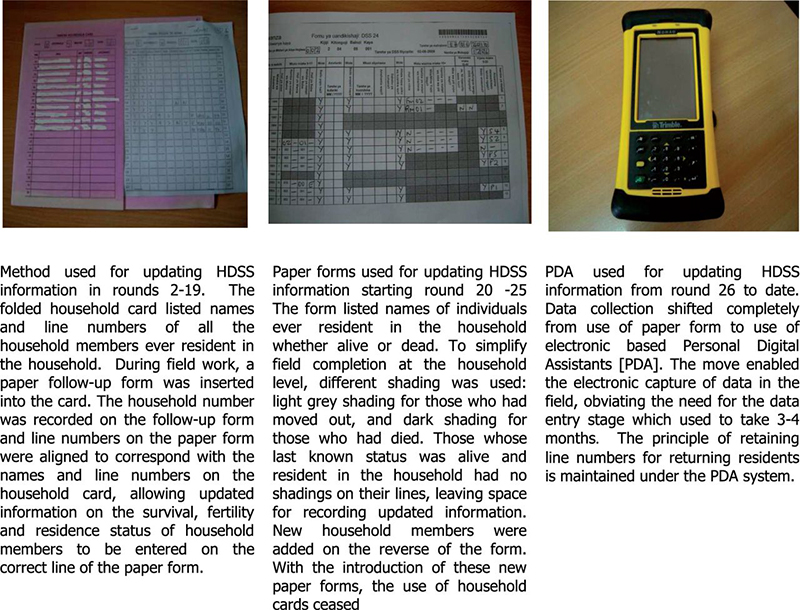
Photoes showing tools used for updating HDSS information.

At the baseline survey in 1994, the population of the area was 19 458 inhabitants, subsequently growing at approximately 2.5–3.0% per year to 35 569 in 2014. Between 1994 and 2014, the Magu HDSS population has been updated 28 times through demographic surveillance follow-up by enumerators who visit all households in the HDSS area. Initially the updates were paper based but, since 2012 (HDSS demographic round 26), the update of the HDSS data have been done electronically using Personal Digital Assistants (PDA) (see Figure 2).

A new person (in-migrant) is listed as a household member if the household respondent had stayed, or was intending to stay, in the household for at least 3 months. Those who left a household in the Magu HDSS and were not expected to return to that household were considered out-migrants from the household but maintained their line number, allowing returning household members to be relisted according to their original HDSS numbers. The main reasons for migration are: marriage, household migration, employment, returning home and schooling.

The population participation rate during demographic surveillance follow-up is 98%. High participation rate of community members in HDSS activities is due to good relationship between the National Institute for Medical Research (NIMR) team, leaders and community members of Kisesa ward.

## What has been measured and how have the HDSS databases been constructed?

**Figure 3.**
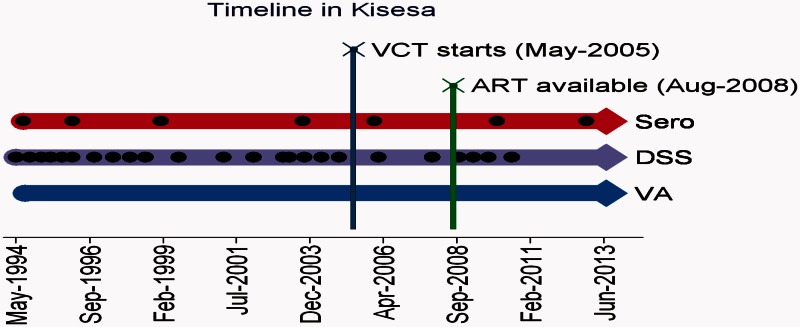
Study timelines and frequency for Magu HDSS Kisesa.

Different data have been measured at different time periods over the past two decades using the Magu HDSS as the framework for the data collection (Figure 3).

### Demographic Surveillance System (DSS)

Following the baseline census conducted in 1994, records are constantly updated. The follow-ups involve visiting every household in the study area and asking one respondent in each household for information on pregnancy, residence, death, in-migration, out-migration, birth, marital status and education (detailed information provided under Table 1) of all residents in the household. The respondents are normally heads of household though, on some occasions, the respondent is another responsible adult household member who is well informed about the household.

**Table 1.** Information currently collected on re-enumeration rounds of the HDSS

Main data item	Scope of question	Specific information
**Household ID**	Each new household	Village number, sub-village number, ten-cell leader number, household number
**GPS status**	Each new household	GPS coordinates
Household reported dissolved	Each household	Yes/no
Is this a new household?	Each household	Yes/no
Line number of household head	Each household	
Line number of respondent	Each household	
**Line number**	All	
**Names**	All	First, middle and last name
**Date of birth**	All	Day, month, year
**Sex**	All	Male/female
**Status at last round**	Existing members	Resident in the household, alive but moved out of household, dead
Still alive	All	Alive, dead
Date of death	Recently dead	Day, month, year
Still resident in this household	All	Yes/no
Date moved out of household	Recently moved out only	Day, month, year
Place moved to	Recently moved out only	Name of place moved to (village code if Kisesa)
Reason for move	Recently moved out only	
Status of new arrival	All new members	Birth, returning previous resident, new in-migrant
Mother’s line number	New members aged 0–17	Line number, or code showing resident in Kisesa, dead, live somewhere outside Kisesa, don’t know
Father’s line number	New members aged 0–17	Line number, or code showing resident in Kisesa, dead, live somewhere outside Kisesa, don’t know
Status of in-migrants	New/returning members only	Resident or visitor
Arrival date	New/returning members only	Day, month, year
Arrival from	New/returning members only	Name of place coming from (village code if Kisesa)
Reason for arrival	New/returning members only	
Slept here last night	All	Yes/no
Marital status	Aged 15+	Never married, married, separated, widowed
Spouse line number(s)	Aged 15+	
Pregnant now	Female aged 15–49 years	Yes/no
Gave birth since last HDSS	Female aged 15–49 years	Yes/no
Still at school	Aged 5–25 years	Yes/no
Type of school	Those at school only	Primary, secondary, tertiary, vocational
Class now attending	Those at school only	Standard number or form number

Data items shown in bold are already available for existing members. They are displayed on the PDA screen and if correct are not re-entered.

### Verbal autopsy

Verbal autopsy (VA) is another important piece of research carried out as part of HDSS work and was initiated in February 1995. During HDSS listing, all deaths encountered are listed on a separate form. The listed deaths are then followed up with a VA interview using the standard WHO/INDEPTH tool for VA. VA interviews are administered by a clinician or clinical officer to the caregiver of the deceased, to establish the possible cause of death. The VA interview collects detailed data on circumstances, symptoms and signs during the terminal illness of the deceased. The collected data are assessed by two trained medical doctors who independently assign the underlying, immediate and contributory causes of death. If the same diagnoses are given by the two doctors, this is accepted as the probable cause of death but, in the case of disagreement, a third medical doctor assesses the data and assigns the causes of death. In addition to VA coding by clinicians, a computer algorithm InterVA-4 has been used to assign the causes of death since 2012.^1^

### Sero survey

Regular HIV sero surveys are another major research activity conducted within the Magu HDSS. All adults aged 15 years or more and living in Magu HDSS have been invited to participate in the survey. During the sero surveys, special temporary clinics are constructed and participants are invited to come to the clinic serving their villages. At the clinic, participants go through six stages: registration, interview, laboratory investigation, treatment, pharmacy and voluntary counselling and testing (VCT). At the interview stage, participants are interviewed on range of topics which include: socio-demographic characteristics, birth history, sexual behaviour, family planning, HIV testing and use of Antiretrovirals (ARVs) (detailed information provided in Table 2). Samples for HIV testing are collected during the laboratory stage. The sample is collected using dry blood spot (DBS) using filter paper. The DBSs are submitted to the NIMR Mwanza Centre for HIV testing using enzyme-linked immunosorbent assay (ELISA). The HIV testing algorithm includes two ELISAs, i.e. UNIFORMII (Vironostika HIV1/HIV2-Organon, Boxtel, The Netherlands) for screening and UNIFORMII (Enzygnost HIV1/HIV2- Behring, Marburg, Germany) for confirmation. Samples reacting positively on the two tests are considered HIV infected. The sero surveys are carried out at 2–3-year intervals and, since 1994, seven rounds have been conducted: 1994/95, 1996/97, 1999/2000, 2003/04, 2006/07, 2010 and 2012/13.

**Table 2.** Information collected at latest questionnaire interview of sero survey round

Subject	Specific information
Background information	Sex, age, marital status, language use, literacy, education level, religion and ethnicity, residence (village, ward), duration of stay in that place
Occupation	Main economic activity for income generation (farming, business, skilled/unskilled manual, fishing, drivers etc.)
Physical activity	Physical activity at work (continuous heavy or vigorous activities like lifting and carrying heavy loads, digging, hoeing, pounding or chopping); travelling (walking, cycling, vehicle use); social activities, (sports etc.)
Food	Consumption of fruit, vegetables and starches in wet and dry seasons
Alcohol use	Frequency, quantity
Tobacco use	Frequency, type, quantity
Condoms	Knowledge, availability
Family planning	Knowledge, uses
Pregnancy and childbirth	Ever been pregnant, ever gave birth, currently pregnant, outcome of last pregnancy, place of delivery, attended ANC during last pregnancy, tested for HIV, syphilis or other blood test during last pregnancy
Desire for children	Want another child yes/no, when, how many more
Marriage	Current marital status, first marriage circumstances
Lifetime sexual behaviour	Age at first sex, condom use at first sex, number of lifetime sexual partners, number of sexual partner in the past 12 months, reasons for abstinence if no sexual partners in past 12 months
Current sexual partners (loop)	Partner details for recent sexual partners: type of partner, cohabitation, age, first and last sex date, frequency of intercourse, condom use
Non-sexual HIV risk factors	Blood transfusion, circumcision, incisions, injections
HIV perception	Awareness, knowledge about transmission, knowledge of infection in family and community, stigma and discrimination among infected
HIV counselling and testing (HTC)	Number of times use HTC, last time use HTC, receive pre/post counselling in last HTC, disclose HIV status, recommend HTC to friend, people’s perceptions about those attending HTC
Anti-retroviral therapy (ART) perceptions	Knowledge, attitude, awareness of use in family and community, local availability
Health services	Use and cost of services
Symptoms of specific illnesses	Whether experienced recent symptoms of sexual transmitted infections, tuberculosis, diabetes, schistosomiasis and hypertension
Care and treatment clinic (CTC) services	Willingness to disclose personal experience of CTC use, ever use, referral
Experience of use of different CTC clinics (loop)	Clinic name, whether tested, assessed for treatment need, received treatment, opportunistic infection treatment, adherence counselling
pre-ART monitoring	Dates and consistency of attendance
ART use	Dates and treatment adherence
Prevention of mother to child transmission for women aged 15–49	HIV test and counselling at the time of delivery, medicine taken at time of delivery, baby given medicine to prevent HIV, baby tested for HIV infection, breastfeeding of the last born

### Voluntary counselling and testing (VCT) and care and treatment clinic (CTC) data collection

VCT services were started at Kisesa Health Centre in 2005 through the collaboration of the TAZAMA Project and Magu District Medical Officer. The VCT clinic offer services to all Kisesa ward residents and sometimes those coming from outside the ward. Those testing HIV-positive in the VCT are referred to the CTC located at the same health facility. The CTC services were initiated at the Kisesa health centre in 2008 through collaboration of the TAZAMA Project and Magu District Medical Officer. The services of both VCT and CTC are delivered according to the guidelines of the Ministry of Health and Social Welfare (MoHSW). Patient information and data on all visits to the CTC by enrolled HIV-positive patients are collected using a national database provided by the National AIDS Control Programme (NACP). The Access Database has data which are entered from the CTC ‘patient record card’. Information collected includes sex, age, marital status, referral status, pregnancies, CD4 count, signs and symptoms, pregnancy status, TB screening, TB treatment, ARV status, care status, ARV adherence, prophylaxis treatment, nutritional status and next visit date

### Qualitative work

Qualitative studies have been used to study perceptions, behaviour, attitudes and practice in Magu HDSS, and to find the reasons why associations found in quantitative studies are seen. Qualitative studies have focused on exploring factors influencing attendance at HIV clinics, antiretroviral therapy scale-up, different types of HIV stigma and voluntary counselling and testing uptake, and perceptions and experiences of barriers to accessing the national antiretroviral programme among self-identified HIV-positive persons. Qualitative studies have been conducted using in-depth interviews (IDI), focus group discussion (FGD) and semi-structured interviews. Interviews and focus group discussions have been conducted among HIV-infected persons, faith leaders, community leaders and health care providers.

### Data management

Since 2010, the HDSS has used electronic data collection (EDC) where Census and Survey Processing System (CSpro) version 4.1 is used to produce computer assisted personal interviewing (CAPI) entry screen for PDA. Then enumerators record household information directly into the PDA. Under this system, households in the study area are organized into batches, with each sub-village representing a batch. The data manager’s office organizes the batches and uploads them onto the enumerators’ PDAs within the CSPro data entry programs. After collection, each interviewer provides the collected data to the HDSS supervisor on a flash disk which is then brought to the HDSS data manager for integration into the Magu HDSS data management system. A batch editing is applied to clean errors and the edited data are stored in the Ms Access and Microsoft Structured Query Language (MS SQL) server databases on the Magu HDSS server.

Sero survey data have also been collected electronically since 2012, and are linked to the HDSS data using the individual’s current household identifier which is pre-printed on the invitation slip for giving to eligible participants. HDSS residents who participate in one or more sero surveys are allocated a unique permanent identification number which links their sero survey records.

## Key findings and publications

**Figure 4.**
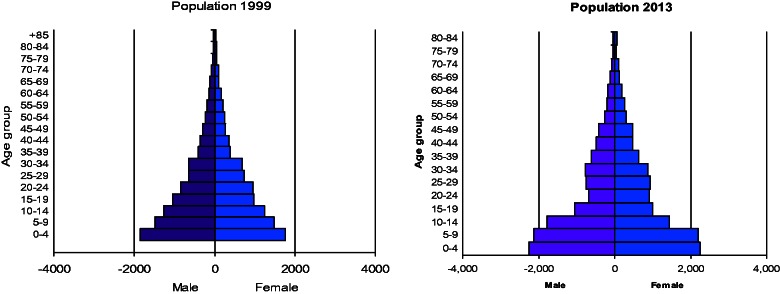
Population pyramid for Magu HDSS.

The population pyramid of the Magu HDSS signifies high fertility and mortality in the study area. The pyramid has a broad base, suggesting that there are a lot births in the cohort and that the population is growing rapidly (Figure 4). There is a marked indentation in the age groups 15–39 for both sexes in 2013 compared with 1999—this may be in part due to HIV-related mortality, but is also a result of increased out-migration by young adults.

**Figure 5.**
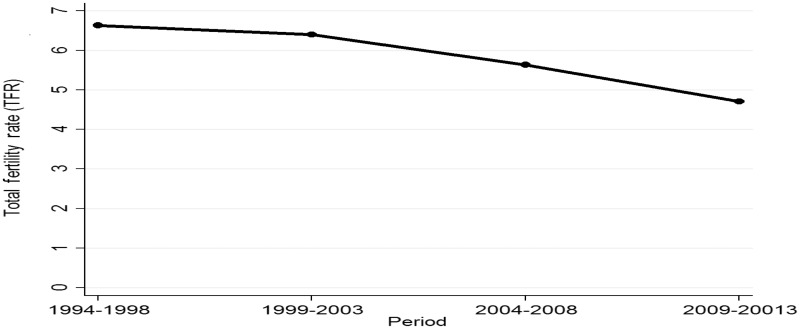
Total fertility rate trends between years 1994 and 2013.

The crude fertility rate fell from 215 [95% confidence interval (CI) 209–222] births per 1000 person-years (PY) in the 1994–98 period to 141 (95% CI 138–145) in 2009–13. In the same period, total fertility rate (TFR) fell from 6.6 to 4.7 births per woman (Figure 5). This is in line with observed changes in fertility in other sub-Saharan African countries.^2^

**Figure 6.**
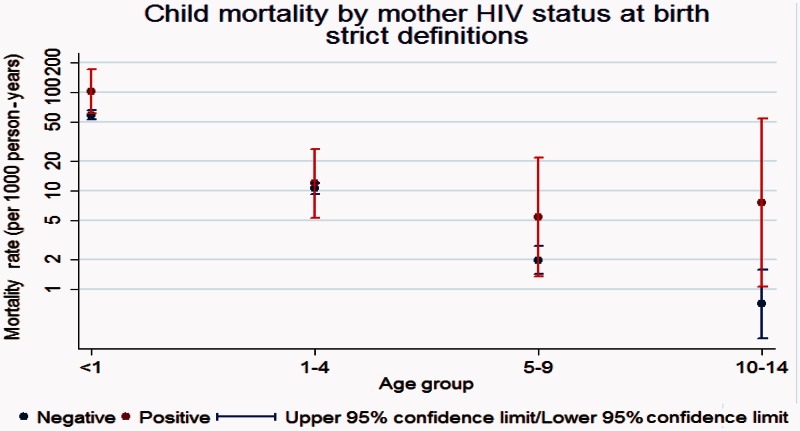
Child mortality by mother’s HIV status at birth.

In the period 1994–2013, child mortality by mother’s HIV status at birth has been high among children born by HIV-positive mothers compared with those born among HIV-negative mothers (Figure 6).

**Figure 7.**
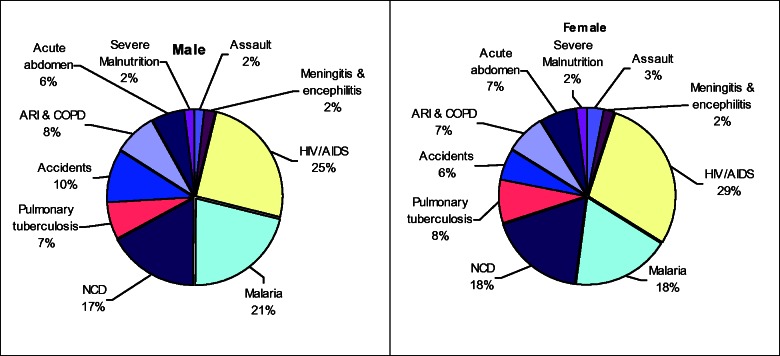
Distribution of major causes of death (all age groups). ARI & COP, acute respiratory infection and chronic obstructive pulmonary disease; NCD, non-communicable disease.

In the assessment of deaths in the area, of those that fall under the top 10 causes of deaths, HIV/AIDS is a major cause of death for all ages, with a higher proportion of deaths among females compared to males. Malaria is the second leading cause of death with more deaths recorded among males compared with females. Pulmonary tuberculosis ranks fourth, with a higher proportion of deaths recorded among females than males. Accidents are the fifth cause of death, with a higher proportion of deaths among males than females (Figure 7).

### Impact of HIV on Mortality

Mortality rates among HIV-infected adults were 15 times higher than those among HIV-negative adults, and almost half of the deaths among those in aged 15–44 years were associated with HIV/AIDS.^3^ Among infants, there was an excess risk of infant death among those born to HIV-positive mothers of 2.9 (95% CI 2.3–3.6) compared with those born to mothers known to be HIV-negative).^4^^,^^5^

Using data from Kisesa and another longitudinal community study in Manicaland in Zimbabwe, we investigated which VA symptoms were most efficient at discriminating between deaths in HIV-positive and HIV-negative individuals. We concluded that verbal autopsy can consistently measure AIDS mortality in areas for which HIV testing and hospital records are unavailable or incomplete.^6^ In the mid 1990s there were a third more deaths due to HIV and AIDS in the Kisesa cohort compared with later years (2004) when ART was available.^7^ Due to the impact of ARV, a substantial mortality decline has been observed among HIV-positive adults, at a time when there was little or no mortality decline in HIV-negative individuals.^8^^,^^9^

### Factors associated with HIV spread

The level of socioeconomic activities based on the presence of main roads, frequency of public transport, numbers of shops, bars and health facilities and volume and frequency of trade plays a big role in the spread of HIV in rural areas of Tanzania.^10^ The trends and patterns in spread of HIV in the HDSS are determined by sexual behaviour.^11–15^

Within families, parental communication about sexual and reproductive health was limited by lack of appropriate knowledge and by cultural norms that restricted open discussion about safe sex and interactions with the opposite sex.^16^

HIV-positive women were more likely to have used family planning, particularly hormonal methods.^17^

### Factors associated with uptake of ARV and VCT

Studies on factors associated with uptake of ARV showed that HIV stigma was the most formidable barrier to accessing the service.^18^^,^^19^ Motivation and self-efficacy contribute to ARV programme retention and are affected by other individual-level experiences such as perceived health benefits or disease severity.^20^ Denial of HIV status was often associated with using alternative healers, and perceptions of illness severity influenced HIV clinic attendance.^21^ Socio-demographic factors, such as marital status, area of residence and religion, influenced VCT completion among males and females in different ways, and self-perceived risk of HIV, prior knowledge of VCT, and sex with a high-risk partner emerged as important predictors of VCT completion among both sexes.^22^ Praying for the sick was a common practice, and over one-third of respondents said that prayer could cure HIV; enhancing faith leaders’ understanding of ART's strengths and pitfalls is an essential step to engaging them as active partners in ART scale-up programmes (Intervention programmes).^23^

VCT services are attracting HIV-infected and high-risk individuals.^24^ However, 2 years after the introduction of antiretroviral therapy, the overall uptake remains low. Among 3923 participants attending two rounds of linked serological surveys (2003–04 and 2006–07), VCT completion in 2006–07 was 17% among the 3702 who were HIV-negative in both rounds, 19% among the 124 who were HIV-infected in both rounds and 22% among the 96 who sero-converted between rounds.^25^

For publications from 1997 to 2008, visit [http://www.tazamaproject.org/] and for publication from 2009 to date, see Appendix 1 (available as Supplementary data at *IJE* online).

## Future analysis plans

Magu HDSS will continue to monitor progression and dynamics of HIV/AIDS in the area, exploring the impact associated with uptake of ARV and VCT on mortality and morbidity in this population. This is especially important given the greater access to HIV services that is currently available in the health facilities, and the need for monitoring the effectiveness of these services. We will perform detailed analysis on morbidity and mortality trends in adults and children and continue using VA to determine causes of deaths in the population while improving the methods used to analyse causes of death using improved versions of InterVA. We will analyse fertility trends in women of child-bearing age and compare fertility by HIV and treatment status to see if there is an upturn in fertility rates when most HIV-infected women receive ART. Finally, we will assess factors associated with uptake of ARV by linking HDSS data with CTC data obtained from health facilities. Collaboration in these and other analyses is welcome, as well as collaborative work in developing new research protocols.

## What are the main strengths and weaknesses?

### Strengths

The move to update HDSS information collection from paper to an electronic personal digital assistance (PDA) system has reduced the processing time for information update, entry and validation. The PDA system has made it possible for the full dataset to be available for use and analysis very soon after the updating and cleaning of the last HDSS records. Thus the HDSS has increased the value of the cohort in conducting studies such as the sero surveys and the linkages to the health facility data.

The study site is currently developing database systems to improve the tracking of individuals’ movements between households within the HDSS area. This will improve migration reconciliation within the HDSS and help to identify former residents when they move back into the Magu HDSS, even if they were outside the area for a long time. They system will also fully link parents and their children.

### Weaknesses

The high cost of running demographic surveillance activities continues to be of great concern, and sometimes activities are delayed due to the lack of funds. Delays in implementing HDSS follow-up rounds are a serious shortcoming, as some events like deaths (especially neonatal deaths), births and short-term mobilities are missed because of time lapse.

## Data sharing and collaborations

Magu HDSS data are held at the NIMR office and are shared through collaborative research agreement with colleagues at the London School of Hygiene and Tropical Medicinewho have contributed to the study designs. Magu HDSS has subscribed to the INDEPTH network’s Sharing and Accessing Repository (iSHARE) data-sharing policy, and our demographic data are lodged in the iSHARE repository. Applications to access HDSS data for collaborative analysis are encouraged and can be made through the project coordinator, Mark Urassa, at [urassamark@yahoo.co.uk] or the director of NIMR Mwanza;], John Changalucha, at [jchangalucha@yahoo.com].

## Supplementary Data

Supplementary data are available at *IJE* online.

## Funding

Since the start of the Magu HDSS there have been three major funders. The first was The Netherlands Government through the TANESA Project (1994–2002). The second major funder was the Global Fund for AIDS, TB and Malaria (GFATM) Round 4 during 2005–10, and this support was renewed in Round 9 (2011–16). The GFATM funding has been arranged through Tanzania’s National Coordinating Mechanism (TNCM). The third major funder has been the Wellcome Trust, which funded all the computerization of data collection. The UK’s Department for International Development (DfID), the Bill and Melinda Gates Foundation (through the ALPHA network) and the US NIH (through the IeDEA consortium) have also contributed to funding certain aspects of the work. The Magu HDSS cohort has received partial funding from several Wellcome Trust grants including the Alpha network grant 090959/Z/09/Z and grant 085477/Z/08/Z.

**Conflict of interest:** None declared.

## Supplementary Material

Supplementary Data
