# Supplementary material for: Health & Demographic Surveillance System Profile: The Magu Health and Demographic Surveillance System (Magu HDSS)
Source: Int J Epidemiol. 2015 Sep 24;44(6):1851–61. doi: 10.1093/ije/dyv188 (PMC4911678; doi:10.1093/ije/dyv188)
Supplement: Supplementary Data [file supp_44_6_1851__index.html]

Health & Demographic Surveillance System Profile: The Magu Health and Demographic Surveillance System (Magu HDSS) — Supplementary Data 

# Health & Demographic Surveillance System Profile: The Magu Health and Demographic Surveillance System (Magu HDSS)

## Supplementary Data

files

- Supplementary Data - pdf file
