# Supplementary material for: Health & Demographic Surveillance System Profile: The Magu Health and Demographic Surveillance System (Magu HDSS)
Source: Int J Epidemiol. 2015 Sep 24;44(6):1851–61. doi: 10.1093/ije/dyv188 (PMC4911678; doi:10.1093/ije/dyv188)
Supplement: Supplementary Data [file supp_dyv188_suppl_data_ije-2015-02-0250.pdf]

Appendix 1: List of TAZAMA project Publication 2009 to date

2009

Busza J, Zaba B, Urassa M. The "Seeded" Focus Group: A strategy to recruit HIV+ community members into treatment research. Sex Transm Infect 2009;85 212-215

Lopman B, Cook A, Smith J, Chawira G, Urassa M, Kumogola Y, Isingo R, Ihekweazu C, Ruwende J, Ndege M, Gregson S, Zaba B, Boerma T. Verbal autopsy can consistently measure AIDS mortality: a validation study in Tanzania and Zimbabwe. J Epidemiol Community Health. 2010 Apr;64(4):330-4. Epub 2009 Oct 23.

Keogh SC, Urassa M, Kumogola Y, Mngara J, Zaba B. Reproductive behaviour and HIV status of antenatal clients in northern Tanzania: opportunities for family planning and preventing mother-to-child transmission integration. AIDS 2009 Nov;23 Suppl 1:S27-35

2010

Wamoyi J, Fenwick A, Urassa M, Zaba B, Stones W. Parent-child communication about sexual and reproductive health in rural Tanzania: Implications for sexual health interventions. 2010 Reproductive Health 7:6

Roura M, Nsigaye R, Nhandi B, Wamoyi J, Busza J, Urassa M, Todd J, and Zaba B. "Driving the devil away": qualitative insights into miraculous cures for AIDS in a rural Tanzanian ward. BMC Public Health. 2010; 10: 427

2012

Raphael Isingo, Alison Wringe, Jim Todd, Mark Urassa, Doris Mbata, Griter Maiseli, Rose Manyalla, John Changalucha, Julius Mngara, Ester Mwinuka, Basia Zaba. Trends in the uptake of voluntary counseling and testing for HIV in rural Tanzania in the context of the scale up of antiretroviral therapy. TM&IH Volume 17, Issue 8, pages e15–e25, August 2012

Alison Wringe, Sian Floyd, Patrick Kazooba, Phyllis Mushati, Kathy Baisley, Mark Urassa, Anna Molesworth, Christina Schumacher, Jim Todd[1], Basia Zaba. Antiretroviral therapy uptake and coverage in four HIV community cohort studies in sub-Saharan Africa. TM&IH Volume 17, Issue 8, pages e38–e48, August 2012.

Milly Marston, Denna Michael, Alison Wringe, Raphael Isingo, Benjamin D. Clark, Aswile Jonas, Julius Mngara, Samweli Kalongoji, Joyce Mbaga, John Changalucha, Jim Todd, Basia Zaba, Mark Urassa. The impact of antiretroviral therapy on adult mortality in rural Tanzania. TM&IH Volume 17, Issue 8, pages e58–e65, August 2012.

Sian Floyd, Milly Marston, Kathy Baisley, Alison Wringe, Kobus Herbst,Menard Chihana, Ivan Kasamba,Till Bärnighausen,Mark Urassa,Neil French,Jim Todd, Basia Zaba . The effect of antiretroviral therapy provision on all-cause, AIDS and non-AIDS mortality at the population level – a comparative analysis of data from four settings in Southern and East Africa. TM&IH Volume 17, Issue 8, pages e84–e93, August 2012.

Sarah C. Keogh, Mark Urassa, Maria Roura, Yusufu Kumogola, Samwel Kalongoji, Daniel Kimaro, John Changalucha, Basia Zaba. The impact of antenatal HIV diagnosis on postpartum

childbearing desires in northern Tanzania: a mixed methods study. Reproductive Health Matters 2012;20(39S):39–49.

2013

Pieter W Smit**,** Thomas van der Vlis**,** David Mabey**,** John Changalucha**,** Julius Mngara**,** Benjamin D Clark**,** Aura Andreasen**,** Jim Todd**,** Mark Urassa**,** Basia Zaba **and** Rosanna W Peeling**.** The development and validation of dried blood spots for external quality assurance of syphilis serology. *BMC Infectious Diseases 2013, 13:102 doi:10.1186/1471-2334-13-102.*

Gourlay A, Birdthistle I, Wringe A, Mshana G, Mkwashapi D, Nsigaye R, Urassa M, Zaba B. Challenges with male involvement in prevention of mother-to-child transmission of HIV services in rural Tanzania: views of fathers, mothers and providers: Oral presentation at the AIDS Impact conference, Barcelona, October 2013.

Gourlay A, Wringe A, Birdthistle I, Marston M, Mkwashapi D, Mshana G, Todd J, Urassa M, Zaba B. Knowledge of vertical transmission of HIV in the context of prevention of mother-to-child transmission of HIV services in rural Tanzania: a mixed methods approach: Poster presentation at the ICASA conference, Cape Town 2013.

2014

Cawley Caoimhe, Wringe Alison, Slaymaker Emma, Todd Jim, Michael Denna, Kumugola Yusufu, Urassa Mark, Zaba Basia. The impact of voluntary counselling and testing services on sexual behaviour change and HIV incidence: observations from a cohort study in rural Tanzania BMC Infectious Diseases.2014, 14:159. DOI: 10.1186/1471-2334-14-159

Kanjala C, Michael D, Todd J, Slaymaker E, Calvert C, Isingo R, Wringe A, Zaba B, Urassa M, Using HIV-attributable mortality to assess the impact of antiretroviral therapy on adult mortality in rural Tanzania. Glob Health Action 2014, **7**: 21865 - http://dx.doi.org/10.3402/gha.v7.21865

Michael D, Kanjala C, Calvert C, Pretorius C, Wringe A, Todd J, Mtenga B, Isingo R, Zaba B, Urassa M. Does the Spectrum model accurately predict trends in adult mortality? Evaluation of model estimates using empirical data from a rural HIV community cohort study in north-western Tanzania. Glob Health Action. 2014;7:doi: 10.3402/gha.v7.24079..

G. Reniers, E. Slaymaker, J. Nakiyingi-Miiro, C. Nyamukapa, A.C. Crampin, K. Herbst, M. Urassa, F. Otieno, S. Gregson, M. Sewe, D. Michael, T. Lutalo, V. Hosegood, I. Kasamba, A. Price, D. Nabukalu, E. Mclean, and B. Zaba, on behalf of the ALPHA Network, Mortality trends in the era of antiretroviral therapy: evidence from the Network for Analysing Longitudinal Population based HIV/AIDS data on Africa (ALPHA)." *AIDS* 28 (2014): S533-S542.

Zaba B, Reniers G, Slaymaker E, Todd J, Glynn J, Crampin AC, Urassa M, Lutalo T, Clark S, Hosegood V, Newell ML, Gregson S. Response to D Maher article "The ethics of feedback of HIV test results in population-based surveys of HIV infection". 2014. Bul WHO. [letter] In Press

Tenu F, Todd J, Urassa M, Isingo R, Zaba B. Adjusting the HIV prevalence for non response using mortality rates in an open cohort in northwest Tanzania. TMIH June 2014. 19:6,656-663 doi:10.1111/tmi.12304

Cawley C, Wringe A, Slaymaker E, Todd J, Michael D, Kumugola Y, Urassa M, Zaba B.

The impact of voluntary counselling and testing services on sexual behaviour change and HIV incidence: observations from a cohort study in rural Tanzania. BMC Infectious Diseases 2014, 14:159

Gourlay A, Wringe A, Birdthistle I, Mshana G, Michael D, Urassa M. “It Is Like That, We Didn't Understand Each Other”: Exploring the Influence of Patient-Provider Interactions on Prevention of Mother-To-Child Transmission of HIV Service Use in Rural Tanzania. PLoS One. 2014 Sep 2;9(9):e106325. doi: 10.1371/journal.pone.0106325. eCollection 2014.
